# A Meta-Analysis of Response Strategies and Interfering Factors of Kin Recognition in Plants

**DOI:** 10.3390/plants14050683

**Published:** 2025-02-23

**Authors:** Xin-Xin Xia, Shaobin Yan, Peng Wang, Chui-Hua Kong

**Affiliations:** 1 College of Resources and Environmental Sciences, China Agricultural University, Beijing 100193, China; waxiaxx@163.com; 2 Institute of Applied Ecology, Chinese Academy of Sciences, Shenyang 110016, China; yanshaobin2022@126.com; 3 University of Chinese Academy of Sciences, Beijing 100049, China

**Keywords:** beneficial plant–plant interactions, biotic and abiotic factors, conspecific cooperation, genetic relatedness, kin discrimination, kinship effect, plant performance

## Abstract

Conspecific plants exhibit morphological and biochemical plasticity in response to genetic relatedness in varying environments. However, the response strategies and factors influencing kin recognition in plants remain unclear. Meta-analysis is an approach to synthesize the effect size of plant–plant and plant–environment interactions. Here, we present the first case of a meta-analysis for response strategies and interfering factors in relatedness-mediated plant–plant interactions. We synthesized the effect of kin recognition on plant performance and environmental factors, based on 104 studies with 4045 cases. As a result, we found that kin recognition reduces root biomass, root length, root–shoot ratio, and lateral root number, lowering belowground competition. Furthermore, kin cooperation enhances aboveground light acquisition by increasing leaf area and boosts reproductive success by increasing seed biomass. The kinship effects are significantly influenced by both biotic (e.g., root interactions, kinship coefficient *r*, sex systems, recognition level) and abiotic factors (e.g., nutrient levels, experiment types, stress type, planting spacing and duration). Our meta-analysis highlights the response strategies and interfering factors of kin recognition in plant performance and environment dynamics, laying the foundation for further research on its ecological evolution and agricultural applications.

## 1. Introduction

Plants can recognize and respond to their neighbor identity by altering growth, reproduction, and defense strategies. Such plant neighbor identity recognition and response may occur in either interspecific or intraspecific interactions among plants [1,2,3]. Recent studies have shown a relatedness-mediated identity recognition within a species, called kin recognition [4,5]. Plant kin recognition refers to the conspecific cooperation that helps plants with genetic relatedness reduce competition and enhance reproduction, ultimately benefiting individuals and the population as a whole [4,6,7]. Such kin recognition has been observed in various types of plant species, ranging from gymnosperms [8,9] to angiosperms [10,11] and from wild species [12,13] to crop plants [14,15] in natural and managed systems. In natural systems, the dispersal range of wild plant seeds is often centered around their mother plant, resulting in clustered offspring from the same mother plant. Therefore, plant kin recognition is relatively common in natural ecosystems and significantly influences population structure and the formation of plant communities. In managed systems, human selection generates crop cultivars, particularly in the case of highly self-pollinating cultivars, which are genetically uniform and have homogenous agronomic traits, whether they are progeny of the same or different mothers [16,17]. Accordingly, kin recognition in crop plants mainly occurs at the cultivar level, as the genetic and morphological uniformity among crop plants is primarily driven by their parents rather than solely by a single maternal source [14,18,19,20].

Plants and their relatives’ interactions involve kin recognition, kin discrimination, and kin selection [17,21]. Plants first recognize kin individuals by integrating environmental cues from neighboring relatives (i.e., kin recognition), then produce differential phenotypic responses (i.e., kin discrimination), and finally, through kin selection, enhance the overall fitness of the population. Kin recognition and discrimination allow plants to optimize survival and reproductive strategies to the composition of their local neighborhood [4,21]. Such cooperative behavior can be promoted through kin selection. Notably, kin recognition is associated with kin discrimination, but kin recognition and kin discrimination are not necessary for kin selection to occur [3,17].

Kin recognition and discrimination can increase the fitness of the entire population by directly regulating the traits of individuals. However, there is no definitive consensus on how plants with specific morphological and biochemical traits respond to kin. Some studies have found that when plants coexist with relatives, the degree of root competition among plants decreases, particularly in root biomass, root length, root surface area, fine root biomass allocation, and the number of lateral roots [8,10,22,23]. However, other studies did not observe this phenomenon [24], and some even reported that root competition was intensified [25]. For example, when offspring of *Cakile edentula* coexist, the fine root biomass is decreased [10], while the root biomass of *Glechoma longituba* is significantly reduced in the presence of relatives [26]. Conversely, in the coexistence of *Impatiens* cf. *pallida* with genetic relatedness, plants exhibit higher root biomass allocation [27]. When *Distichlis spicata* grows in the root exudates of kin, its number of lateral roots and root length are significantly greater than when it grows in the root exudates of non-kin [28].

The coexistence of conspecific plants with genetic relatedness affects not only belowground traits but also aboveground traits and reproductive performance. For instance, when kin coexist, leaves are reoriented to reduce competition for light resources [29]. Additionally, some plants, such as *Oryza sativa* and herbicide-resistant barnyardgrass (*Echinochloa crus-galli* L.), showed significantly faster flowering time and higher aboveground biomass and yield when coexisting with kin compared to non-kin [13,19,30]. These differences may arise from the inherent growth characteristics of plants and variations in experimental procedures, but the impact of kin recognition on plant performance traits is exploring.

Plants can detect and respond to complex environmental cues, adjusting their responses accordingly [31,32,33]. Biotic and abiotic signals, such as spectral cues, aerial volatiles, root exudates, and mycorrhizal networks, are key pathways through which plants detect the presence of kin and non-kin individuals [29,34,35,36]. When plants grow alongside their kin, leaf positioning may adjust to reduce competition for light resources [29]. Aerial volatile signals can reduce herbivory from insect herbivores on kin plants [7]. When plants are exposed to root exudates from kin, root biomass is significantly reduced, thereby decreasing the intensity of belowground competition among relatives [35]. Additionally, increased investment in mycorrhizal networks between kin enhances the efficiency of nutrient uptake and utilization among related individuals [36]. Furthermore, kin recognition is regulated not only by single factors but also by the combined effects of biotic and abiotic factors. Biotic factors, including root interactions [13,37], kinship coefficient [38], sex systems [28], and the level of kin recognition [39], and abiotic factors, such as planting density [24], nutrient availability [40,41], pot size [42], and planting duration [43], can significantly affect kin recognition and discrimination. Biotic factors determine the likelihood of kin coexistence, while abiotic factors influence the level of resource competition. Therefore, beyond examining the direct impact of kin recognition and discrimination on overall plant performance, exploring the underlying factors that interfere with kinship effects is essential to deepening our understanding of relatedness-mediated intraspecific interactions in plants.

Kin recognition in plants is a complex ecological process. Although many studies have explored the response traits and factors influencing kinship effects, there is still a lack of comprehensive quantitative analysis of response strategies and interfering factors in relatedness-mediated intraspecific plant–plant interactions. Accordingly, the goal of this study is to examine the effect size of kin recognition on plant morphological plasticity and further explore the factors contributing to the kinship effect by means of a meta-analysis. We aim to lay the foundation for future studies enabling increased plant performance under kin recognition and discrimination in plants.

## 2. Results

### 2.1. Phenotypic Response of Kin Recognition in Plants

**Figure 1:**
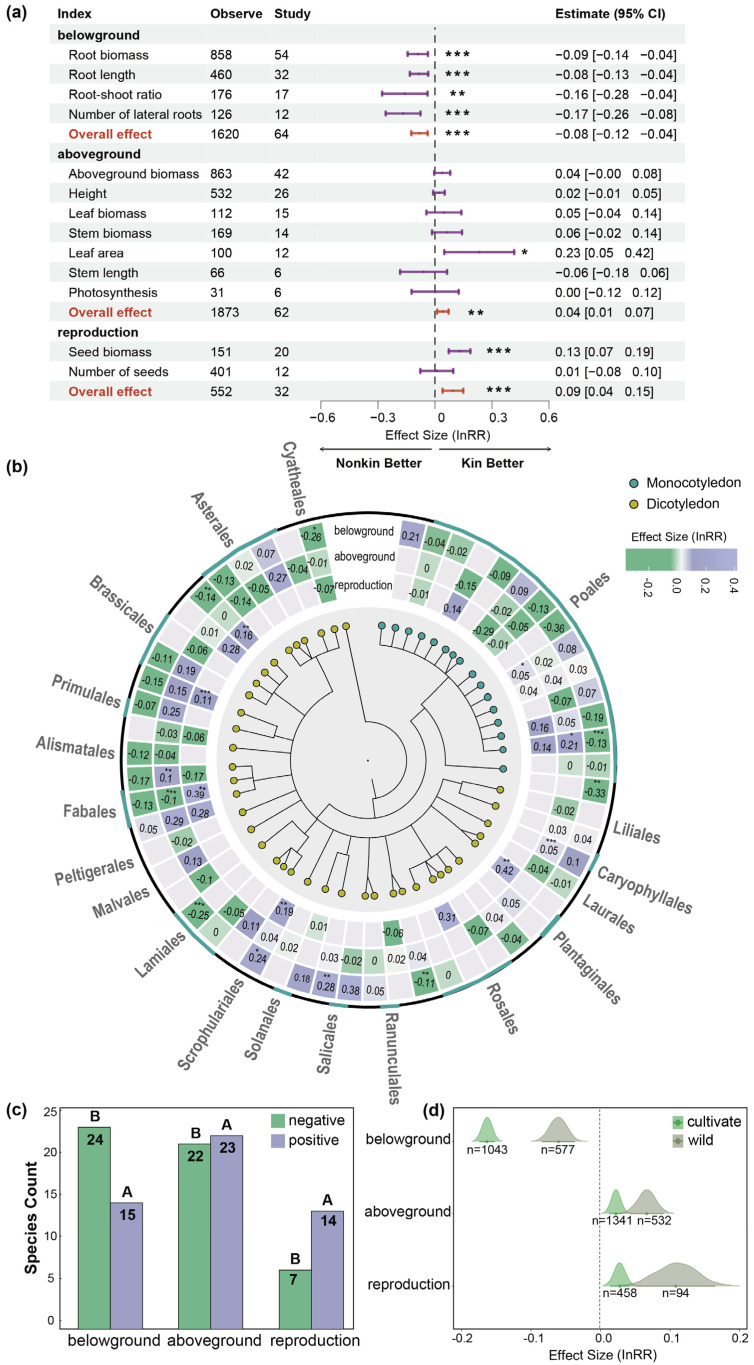
The effects of kin recognition on plant performance. (**a**) The influence of kin recognition on aboveground, belowground, and reproductive traits. The estimated values and 95% confidence intervals for each indicator, as well as observed values (Observe) and the number of studies (Study), are presented. (**b**) The impact of phylogenetic relationships on kin recognition. The phylogenetic relationships of the studied plants are incorporated, with numbers representing cumulative effect values (Effect Size InRR); the outer circle shows the effect values for belowground performance, the middle circle for aboveground performance, and the inner circle for reproductive performance. (**c**) The number of species involved in the positive (purple) and negative (green) effects of kin recognition on belowground, aboveground, and reproductive responses. (**d**) The effects of cultivated crops (green) and wild plants (brown) on kin recognition performance. The plants are categorized into crops and wild plants, with cumulative effect values calculated for their aboveground, belowground, and reproductive performance. A mixed-effects model was used for the analysis, and the restricted maximum likelihood (REML) method was employed to estimate the variance components. In the results, asterisks indicate the significance level: *** *p* < 0.001, ** *p* < 0.01, and * *p* < 0.05.

Throughout the literature, plants exhibit poor belowground performance when coexisting with kin (InRR = −0.08, 95% CI = [−0.12, −0.04]), as indicated by decreases in root biomass, root length, root–shoot ratio, and lateral root number under kin coexistence. Meanwhile, their aboveground performance (InRR = 0.04, 95% CI = [0.01, 0.07]) and reproductive performance (InRR = 0.09, 95% CI = [0.04, 0.15]) demonstrated better outcomes in the presence of kin, particularly in leaf area and seed biomass (Figure 1a). Funnel plot test results indicate that, except for the reproductive response during the coexistence of related plants (z = −0.9337, *p* = 0.3504), there was a certain degree of publication bias in both aboveground response (z = −2.0918, *p* = 0.0365) and belowground response (z = 5.9890, *p* < 0.01). The fail-safe N for plant kin recognition responses in belowground (N_Fail-safe_ = 8,995,140), aboveground (N_Fail-safe_ = 460,313), and reproductive responses (N_Fail-safe_ = 96,432) were all much greater than 5K + 10, indicating that our analysis results were highly robust (Figure S1).

There were no significant differences in the responses of monocotyledonous plants and dicotyledonous plants when coexisting with their relatives based on phylogenetic information (Figure 1b). However, plants within the same genus or family, which are genetically closer according to phylogenetic relationships (e.g., Order, Family, Genus, Species), exhibited similar response traits when coexisting with their relatives (Figure 1b). In terms of aboveground and reproductive performance, most plant species performed better when kin coexisted. However, the opposite trend was observed belowground, where most species showed poor performance, as indicated by smaller root biomass, shorter root length, lower root–shoot ratio, and fewer lateral roots in the presence of kin (Figure 1c; Table S1). There were significant heterogeneities in the responses of plant performance to the presence of kin between crop cultivars and wild species, although the directions of responses were consistent (Figure 1d). For belowground performance, the effect size in cultivated crops (lnRR = −0.163; *p* < 0.05) was more negative than that in wild species (lnRR = −0.061; *p* < 0.05), indicating that crops experienced a greater decrease in performance compared to wild species when coexisting with kin.

### 2.2. Factors Affecting Belowground Responses

**Figure 2:**
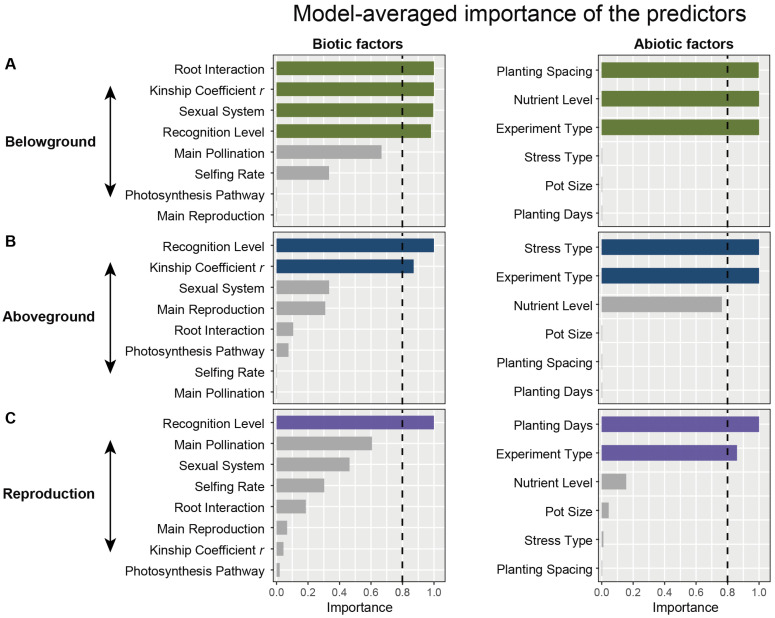
Biotic and abiotic factors affecting kin recognition and kin discrimination in plants. The average importance of biotic and abiotic predictive factors on the log response ratios of kinship effects on (**A**) aboveground, (**B**) belowground, and (**C**) reproductive performance, based on averaged models using AICc (corrected Akaike information criterion).

Biotic and abiotic factors significantly affected responses of plant performances to plants coexisting with their kin neighbors (Figure 2; Table S2). The belowground performance of plants in kin interactions was significantly influenced by factors such as root interaction types, kinship coefficient r, sexual system, kin recognition level, planting spacing, nutrient levels, and experiment type (Figure 2A). In contrast, aboveground performance was primarily affected by kin recognition level, kinship coefficient r, stress type, and experiment type (Figure 2B). Reproductive performance was mainly influenced by kin recognition level, planting duration and experiment type (Figure 2C).

**Figure 3:**
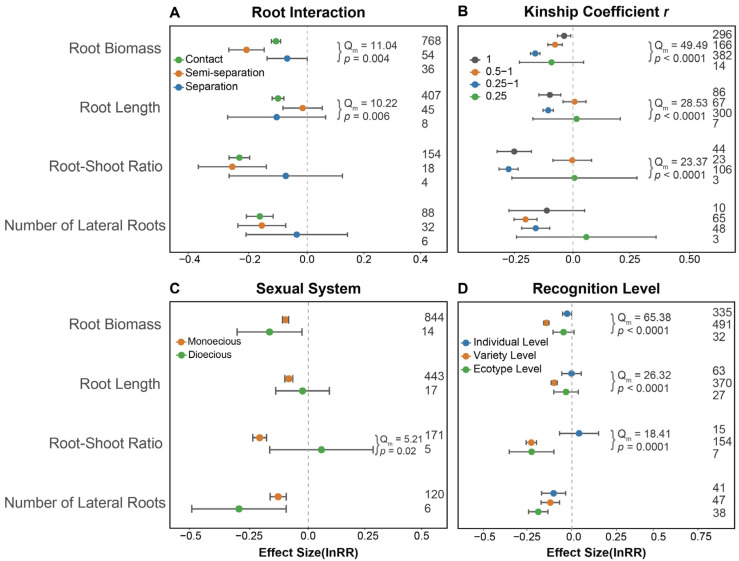
Biotic factors influencing the belowground responses to kin coexistence in plants. (**A**) The effect of root interactions on belowground performance under kin coexistence. (**B**) The kinship coefficient *r,* representing the genetic distance between plants. (**C**) The effect of plant sexual system on belowground performance during kin coexistence. (**D**) The effect of plant kin recognition level on belowground performance during kin coexistence. Digitally represented observations.

Further subgroup analysis revealed significant variation in root biomass and root length under different root interaction types during kin coexistence (Figure 3A). When roots were in contact, kin coexistence led to a significant decrease in root biomass and root length. However, when plant roots were separated, this effect disappeared (Figure 3A). The kinship of coexisting species, which is described as kinship coefficient *r* with a larger value indicating closer kinship and genetic similarity between two individuals, showed that root biomass, root length, and root–shoot ratio responded diversely to plant kin coexistence. Specifically, as the kinship coefficient *r* increased, the response of root biomass became weaker under kin coexistence (Figure 3B). Monoecious plants responded to kin coexistence through belowground traits more than dioecious plants (Figure 3C). Root biomass, root length and root–shoot ratio responded varyingly to the level of plant kin recognition (specific criteria for the classification of plant kin recognition levels are shown in Table S2). Root biomass decreased more drastically at the varietal level than individual level. Root length only responded negatively at the varietal level but had no significant response at the individual level and ecotype level, while root–shoot ratio reduced observably at the varietal level and ecotype level (Figure 3D).

**Figure 4:**
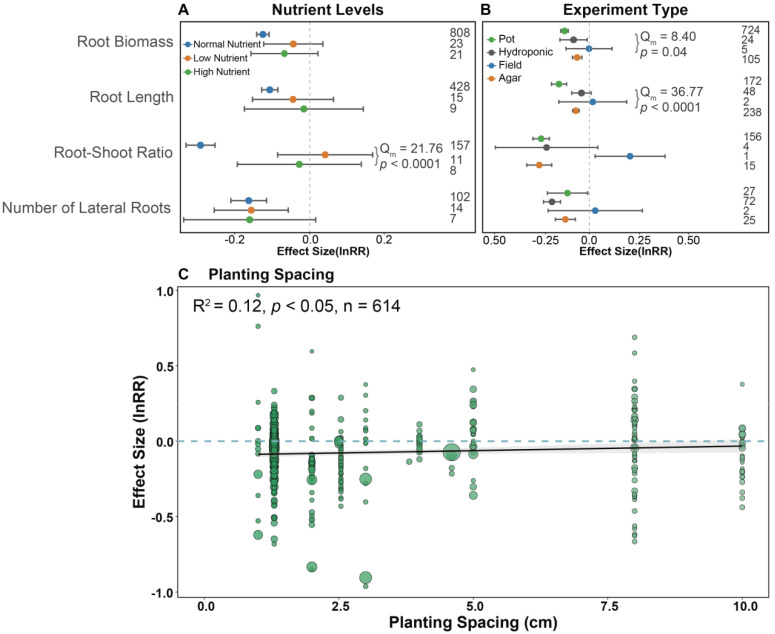
Abiotic factors influencing the belowground responses to kin coexistence in plants. The effect of (**A**) nutrient levels and (**B**) experiment types on belowground performance under kin coexistence. (**C**) Planting spacing is based on the methods section of the included literature; the size of the green dots represents the weight of each factor, with larger dots indicating greater weight. We also show the estimated effect curve along with its 95% confidence interval. Digitally represented observations.

Nutrient levels significantly affected the root–shoot ratio, and there were similar responses of root biomass, root length and number of lateral roots to plant kin recognition under different nutrient levels. Root length responded markedly to kin under a normal nutrient level; however, this response disappeared under low or high nutrient levels (Figure 4A). The decrease in root biomass and root length varied remarkably among experiment types, especially in the pot experiment and agar experiment (Figure 4B). Moreover, the effect size of belowground performance significantly increased with increasing planting spacing, i.e., positive correlation (Figure 4C).

### 2.3. Factors Affecting Aboveground Responses and Reproductive Performance

**Figure 5:**
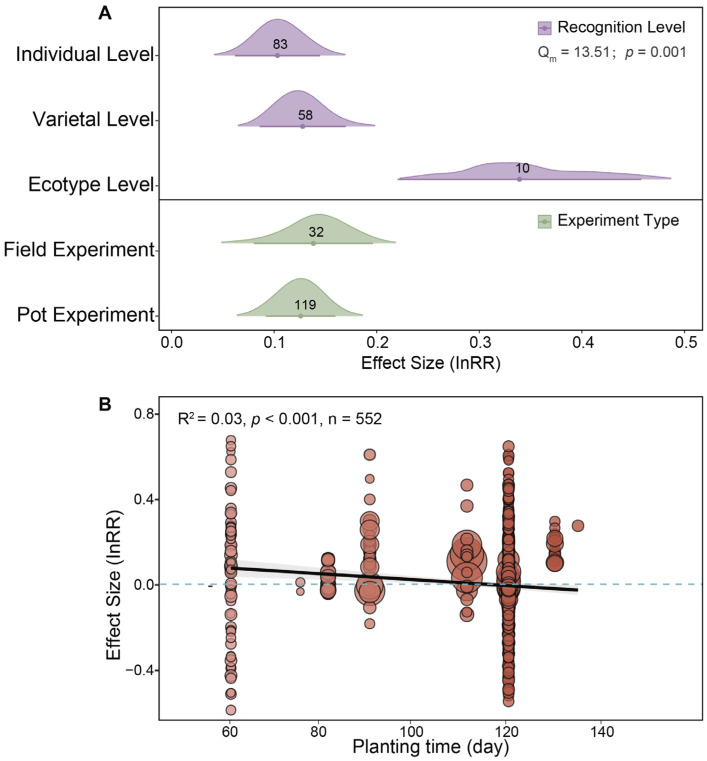
Factors influencing seed biomass under plant kin coexistence. (**A**) The impact of kin recognition level and experiment type on seed biomass during kin coexistence. (**B**) The effect of planting duration on seed biomass during kin coexistence. The size of the red dots represents the weight of each factor, with larger dots indicating greater weight. We also show the estimated effect curve along with its 95% confidence interval. Digitally represented observations.

The leaf area was significantly affected by kin recognition level among plants (Figure S2). Leaf area increased more highly at the ecotype level than at the individual level. Furthermore, the leaf area significantly increased under high kin coefficient *r* (i.e., *r* = 1, *r* = 0.5–1), non-stress conditions, and pot experiments (Figure S2). Importantly, seed biomass at the individual, variety, and ecotype levels was significantly higher than that of non-kin (Figure 5A). There was no difference in the seed biomass response between field and pot experiments. Furthermore, the duration of planting was negatively correlated with the reproductive effect value (InRR) when plants coexisted with their kin. In the early stages of plant maturity, reproductive performance among kin was superior; however, as planting duration increased, the reproductive advantage of kin gradually diminished, leading to reduced differences in reproduction between kin and non-kin coexistence over time (Figure 5B).

### 2.4. Structural Equation Model Analysis for Effects of Kin Recognition on Plant Performance

**Figure 6:**
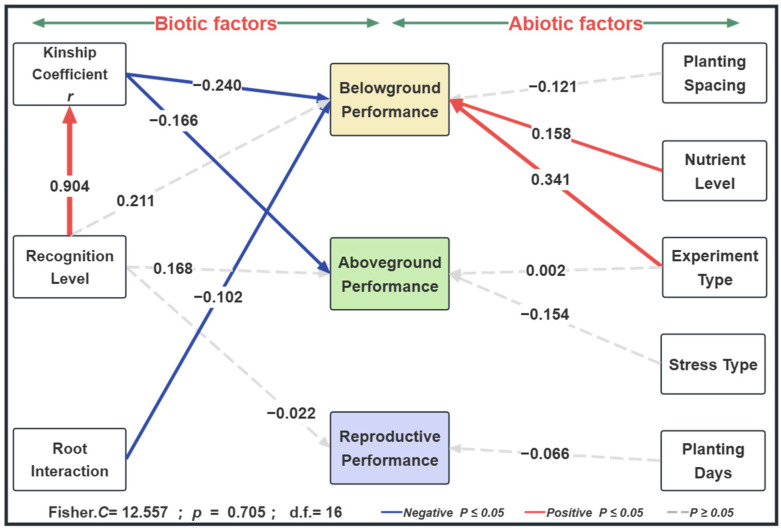
Structural equation modeling analysis of the impact of kin recognition on plant performance. The numbers next to the arrows in the structural equation model represent path coefficients, while the width of the arrows indicates the strength of the correlations. Blue arrows represent negative correlations and red arrows represent positive correlations; solid lines indicate significant relationships (*p* < 0.05), while dashed lines indicate non-significant relationships (*p* ≥ 0.05).

Root interaction, kinship coefficient ***r***, nutrient level, and experiment type directly affect belowground performance among relatives (Figure 6). As the degree of root contact increased (from fully separated to semi-separated, and then to fully connected), the level of belowground competition among kin decreased (standardized regression coefficient = −0.102). Higher kinship coefficients ***r*** between plants also significantly reduced belowground competition during kin coexistence (standardized regression coefficient = −0.240). Nutrient level and experiment type showed different patterns: with increasing nutrient levels and as the experiment type transitioned from hydroponic to larger-scale field experiments, the belowground performance during kin coexistence was significantly higher than during non-kin coexistence (standardized regression coefficient for nutrient level = 0.158, standardized regression coefficient for experiment type = 0.341). Level of kin recognition indirectly impacted belowground and aboveground performance via affecting the kinship coefficient *r* (standardized regression coefficient = 0.904). Additionally, the kinship coefficient *r* during kin coexistence also significantly affected aboveground performance. As the kinship coefficient *r* increased, the level of aboveground competition among plant kin decreased during coexistence (standardized regression coefficient = −0.166) (Figure 6 and Figure S3).

## 3. Discussion

Meta-analysis is an approach to synthesize the effect size of plant–plant and plant–environment interactions, such as the effect of allelopathy on plant performance in plant–plant interactions [44] or the effects of ground-based extraction technologies on fine roots in forest soils [45]. Our meta-analysis makes the first case for relatedness-mediated plant–plant interactions. From a meta-analysis of 104 studies comprising 4045 cases, we found consistent results that kin recognition can improve plant performance by changing belowground traits (root biomass, root length, root–shoot ratio, and number of lateral roots), aboveground traits (leaf area), and reproductive traits (seed biomass) (Figure 1a). However, this improvement is influenced by biotic factors including root interactions, kinship coefficient *r*, kin recognition level, and sexual system, and abiotic factors involved in planting spacing, nutrient levels, experiment type, stress type and planting duration (Figure 2)

Kin recognition is a conspecific cooperation among plants, allowing behaviors toward kin groups that promote the survival and reproduction of relatives. Reducing the energy devoted to competitive root systems allows for greater allocation to reproduction in kin groups [46,47,48]. In terms of root-related metrics, compared to coexistence with non-kin, plants show significant reductions in root biomass, root length, root–shoot ratio and number of lateral roots when coexisting with kin, leading to a substantial decrease in belowground performance overall (Figure 1a). This phenomenon indicates a weakening of belowground competition among relatives. However, during kin coexistence, plants enhance their aboveground growth capacity by increasing leaf area, exhibiting a trend of energy allocation skewed towards aboveground parts. In terms of reproductive performance, kin coexistence significantly increases seed biomass (Figure 1a and Figure 5). Notably, both cultivated crops and wild plants exhibit decreased belowground responses and increased aboveground and reproductive responses when coexisting with kin. However, cultivated crops display a more pronounced reduction in belowground competition when coexisting with kin (Figure 1d).

Kin recognition level is a key factor determining the performance of kinship interactions. It not only affects belowground performance but also influences aboveground growth and reproductive outcomes. Wild plants typically use sibling offspring or the same ecotype as kin [13,43,49], while crop plants often utilize closely related cultivar as relatives [15,18,50]. Meta-analysis indicates that kin recognition at the crop cultivar level can significantly reduce belowground competition and enhance reproductive performance among kin groups (Figure 3D and Figure 5A). Root behavior and plasticity allow plants to adapt to their local environment. Kin recognition and discrimination can result in differences in root growth and placement [15,47,48]. Meta-analysis also found that the degree of root contact between kin affected belowground kinship effects (Figure 3A). When roots came into contact, the intensity of belowground competition (root biomass, root length, root–shoot ratio and number of lateral roots) between kinship plants decreased, lowering competition among them.

The relatedness coefficient *r* reflects the kinship distance, which significantly influences both the aboveground and belowground performance of interacting plants (Figure 2A,B). Meta-analysis indicates that a higher relatedness coefficient leads to less belowground competition among relatives (Figure 3B). The sexual system of self-fertilization in plants also affects the probability of kin interactions among offspring [38]. This meta-analysis shows that the sexual system significantly impacts belowground interactions between kin plants (Figure 2A and Figure 3C).

Biotic factors and abiotic factors are important external conditions that affect the performance of kin recognition in plants [24,29,40,51]. Through meta-analysis, we found that the outcomes of kin recognition were significantly influenced by nutrient resources, environmental stress, and experiment types (Figure 2). Nutrient resources play a crucial role in performance of closely related plants. Nutrients not only directly affect plant growth but also shape plant population dynamics through complex competition and cooperation. When nutrients are at normal levels (without any addition or reduction), belowground cooperative effects among closely related plants are more pronounced. However, in resource-poor environments, competition for limited nutrients among closely related plants reduces the effectiveness of kin recognition (Figure 4A). Plant responses to nutrients are also influenced by the type of experiment. In pot experiments, belowground performance declines (Figure 4B), while aboveground performance increases under kin coexistence (Figure S2). In contrast, under field conditions, kin recognition does not significantly impact belowground performance but can enhance reproductive benefits, particularly by increasing seed biomass (Figure 4B and Figure 5A). These results indicate that kin coexistence in plants is influenced by multiple external factors, which together determine cooperative strategies to maximize resource utilization.

Of note, we further conducted a publication bias test using both a funnel plot and fail-safe N (Figure S1). The results suggest that in studies of kin recognition in plants, particularly when analyzing aboveground and belowground responses, publication bias may be present. Such bias could lead to distortions in meta-analysis results, thereby affecting our understanding of the effects of kin coexistence in plants. Future studies should consider how to reduce publication bias in their design, ensuring that all relevant results are included in the analysis, thus enhancing the reliability and validity of the research.

## 4. Materials and Methods

### 4.1. Literature Search and Identification

Literature was retrieved from three major databases: Web of Science (https://www.webofscience.com), and Google Scholar (https://scholar.google.com), and China National Knowledge Infrastructure (CNKI) (https://www.cnki.net/), using the keywords “plant kin recognition”. The search cutoff date was January 31, 2024, and the process is outlined in Figure S4. The following criteria were established for literature selection: (1) the studies aimed to investigate the effects of plant kin recognition and phenotypic traits (the specific traits are shown in Figure S5) of both kin and non-kin groups; (2) the phenotypes of the kin group were considered the treatment group, while those of the non-kin group served as the control group; (3) experiments included replicates and the studies reported the sample sizes (n), means, standard deviations (sd), or standard errors (se) for both treatment and control groups.

After search and screening, a total of 104 studies comprising 4045 cases were identified. The research locations included 17 countries, primarily concentrated in Asia, Europe, and North America (Figure S6). For each study, we extracted the mean, standard deviations (sd) or standard errors (se), and sample size for the corresponding indicators of kin and non-kin groups from tables or figures. If statistical metrics were not reported in table or graphical form, studies were included if the authors could provide original data or access to download the data. Given that data from some studies was represented in graphical form, digitizing software GetData Graph Digitizer 2.25 (http://getdata-graph-digitizer.com) was used to extract results in figures. The standard errors (se) were not applied directly; we could obtain the corresponding standard deviations (sd) following the formula: $sd=se\sqrt{n}$, where ‘n’ is the corresponding sample size. If one kin group treatment corresponded to different non-kin groups, all measurements were recorded and treated as independent observations. Multiple treatments—for example, different root interactions, kin recognition level, nutrient level, stress type, planting spacing, sampling time—were also recognized as independent observations.

We categorized the research indicators into three classes: belowground responses, aboveground responses, and reproductive responses (Figure S5). We also considered the impact of plant kin recognition on photosynthesis, including net photosynthetic rate, chlorophyll content, stomatal conductance, instantaneous water use efficiency, and transpiration rate. Given that plant kin recognition is influenced by both biotic and abiotic factors, we further incorporated eight biotic factors (root interaction, kinship coefficient *r*, sexual system, recognition level, main pollination, selfing rate, photosynthesis pathway, main reproduction) and six abiotic factors (planting spacing, nutrient level, experiment type, stress type, pot size, planting days) for subgroup analyses and meta-regression to explore the effects of these factors in greater depth (Table S2). The biotic and abiotic factors used in this study were derived from the information provided in the 104 selected articles. We considered pot size an abiotic factor because pot size affected the competition and cooperation between plants.

### 4.2. Calculation of Effect Size (lnRR) and Variance (*V_i_*)

The effect size for individual study cases was calculated using the log response ratio (lnRR) [52], and the corresponding within-case variance (*V_i_*) was computed, where *Y_e_* represents the mean value of the kin group and *Y_c_* represents the mean value of the non-kin group. In addition, the construction of the phylogenetic tree for plants was based on taxonomic information (family and genus) from the research materials included in the referenced literature (Table S1).

$$InRR=In\frac{Y_{e}}{Y_{c}}$$

The sampling error variance of the lnRR was calculated as:

$$V_{i}=\frac{S_{e}^{2}}{N_{e}Y_{e}^{2}}+\frac{S_{c}^{2}}{N_{c}Y_{c}^{2}}$$

where *S_e_* represents the standard deviation of the treatment, *N_e_* represents the sample size of the treatment, and *Y_e_* represents the mean value of the treatment. *S_c_* represents the standard deviation of the control, *N_c_* represents the sample size of the control, and *Y_c_* represents the mean value of the control.

### 4.3. Hierarchical Models, Model Averaging, and Structural Equation Modeling

When analyzing the impact of kinship on plant performance, considering the hierarchical dependency of data across different studies, a hierarchical nested model was employed to estimate the overall effect size of plant kin recognition, using the rma.mv function from the metafor package [53]. The ‘study’ was set as random factor, because some studies contributed an additional effect size, in which multiple independent observations were included in one study. Maximum likelihood estimation was used to calculate weight effect size (lnRR) and 95% confidence interval (95% CI) for each variable. A significant response of a variable was considered when 95% CI of a given variable did not overlap zero (*p* < 0.05). A threshold of 0 was used; if the lnRR value and its 95% confidence interval were less than 0, it indicated that kin recognition reduces plant performance, while the converse indicated that it promoted plant performance.

To explore the key factors influencing the effects of kin recognition in plants, the best combination of explanatory variables was selected using the corrected Akaike Information Criterion (AICc). A lower AICc value indicates better model performance in balancing goodness of fit and complexity. Model averaging was conducted in R using the glmulti package [54], and then the sum of Akaike weights for the models was calculated for each variable as relative importance value. A threshold of 0.8 was used to distinguish between relatively important factors and unimportant factors. After selecting factors with importance greater than 0.8 using model averaging, a mixed-effects model was used for analysis. The construction of the mixed-effects model was as follows:

$$Q_{m}=\sum_{j=1}^{p}w_{j}{\left(\bar{y_{j}}-\bar{y}\right)}^{2}$$

$$\mu_{mix}=\frac{\sum w_{i}\cdot y_{i}}{\sum w_{i}}$$

where *p* is the number of studies, *w_j_* is the weight of the *j*-th study, *ȳ_j_* is the effect size of the *j*-th study, *ȳ* is the weighted average effect size across all studies, and *w_i_* is the weight that accounts for both fixed and random effects. The estimation of the between-study variance τ^2^ was performed using the Restricted Maximum Likelihood (REML) method.

To investigate the impact of important factors (factors with importance greater than 0.8) on plant kin coexistence, we employed two methods: (1) For categorical variables, subgroup analysis was conducted to assess the effect size of each factor on plant kin recognition. A *p* < 0.05 indicated a significant difference between groups; (2) Meta-linear regression analysis was used to determine the relationship between continuous variables and plant kin coexistence, and assign appropriate weights based on different research cases. The formula for weight calculation was:

$$W_{i}=1/\sqrt{V_{i}}$$

Structural Equation Modeling (SEM) can be used to assess the effects of multiple causal relationships and latent variables [55]. We constructed a structural equation model to explore the direct and indirect factors influencing plant kin recognition. We used the psem function from the piecewiseSEM package in R to build the structural equation model [56]. To develop an integrated theoretical framework for plant kin interactions, we considered both biotic and abiotic factors, selecting those with model-averaged importance greater than 0.8. We then used structural equation models to explore the influence of these factors on the performance of plant relatives, examining their effects on belowground, aboveground, and reproductive performance, respectively.

### 4.4. Publication Bias Test

A funnel plot was used to assess the presence of publication bias in the study results. The more symmetrical the plot, the less likely the results are to be affected by publication bias, indicating higher reliability of the study findings (Figure S1).

We further used the Fail-safe N to assess the robustness of the meta-analysis results [57]. Fail-safe N reflects the number of non-significant studies that would need to be added in order to nullify the current significant result. A larger Fail-safe N indicates higher robustness of the results. Typically, we used 5k + 10 as a threshold, meaning that if N_fail-safe_ > 5k + 10 the meta-analysis results are considered robust, where k represents the number of studies.

## 5. Conclusions

The importance of relatedness-mediated plant–plant interactions within a species in fitness-determining processes such as performance, survival, reproduction and resource capture cannot be overemphasized. Recent efforts have made considerable progress toward understanding kin recognition and kin discrimination in intraspecific plant–plant interactions. Nevertheless, much remains unknown. In particular, a few studies have been criticized for flaws in experimental design and the problem of contradictory results. Our meta-analysis of plant performance and environmental factors of kin recognition could account for these controversial issues, providing basic response traits and environmental factors for further understanding and experimental design. Importantly, kin recognition and kin discrimination are beneficial plant–plant interactions. In particular, kin recognition can occur at the crop cultivar level, and significantly enhance reproductive performance among kin groups. Accordingly, crop plants are predicted to increase yield by reducing intraspecific competition and shifting resource allocation to reproduction. Preferentially reducing competitive effects on relatives may improve our understanding of the processes at play in kin recognition to develop cultivar mixtures that increase grain yields in the limited areas suitable for agriculture.

## Data Availability

All data supporting the findings of this study are available within the paper and within its Supplementary Materials published online.

## Supplementary Materials

The following supporting information can be downloaded at: https://www.mdpi.com/article/10.3390/plants14050683/s1, Figure S1. Publication bias tests for kin recognition on aboveground, belowground, and reproductive performance. Figure S2. Biotic and abiotic factors influencing leaf area responses during kin coexistence in plants. Figure S3. Standardized effect sizes of biotic and abiotic factors. Figure S4. The literature screening process for the meta-analysis included in this study. Figure S5. Classification of response metrics for kin recognition in plants. Figure S6. Study locations included in this meta-analysis. Table S1. Study materials included in the literature on plant kin recognition and their aboveground, belowground, and reproductive performance when coexisting with kin. Table S2. Levels of interfering factors included in the meta-analysis of kin recognition in plants.
