# Supplementary material for: A Meta-Analysis of Response Strategies and Interfering Factors of Kin Recognition in Plants
Source: Plants (Basel). 2025 Feb 23;14(5):683. doi: 10.3390/plants14050683 (PMC11901976; doi:10.3390/plants14050683)
Supplement: Supplementary file 1 [file plants-14-00683-s001.zip › plants-3468325-supplementary.pdf]

Supplementary materials

A meta-analysis of response strategies and interfering factors of kin recognition in plants

Xin-Xin Xia [1], Shaobin Yan [2,3], Peng Wang [2,*] and Chui-Hua Kong [1,*]

This supplementary material was prepared to add the readers more details, for which there was not enough space in the main manuscript, about:

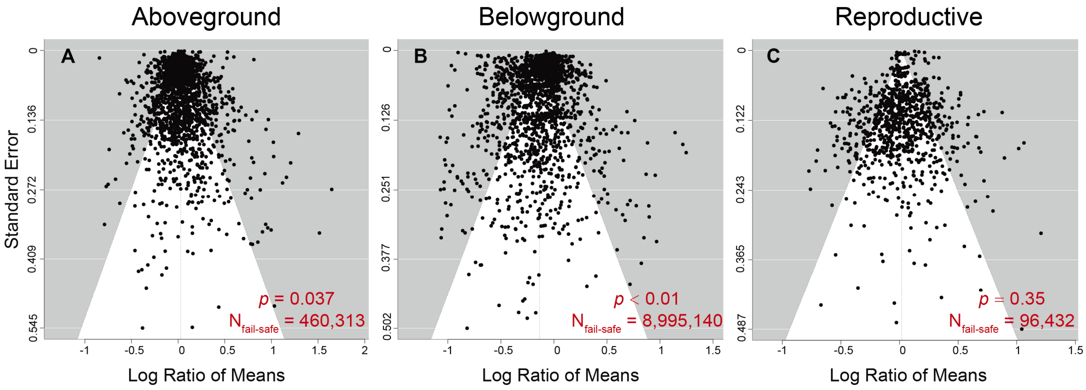

Figure S1. Publication bias test for plant kin recognition was conducted using funnel plots and fail-safe N to assess the aboveground response (**A**), belowground response (**B**), and reproductive response (**C**).

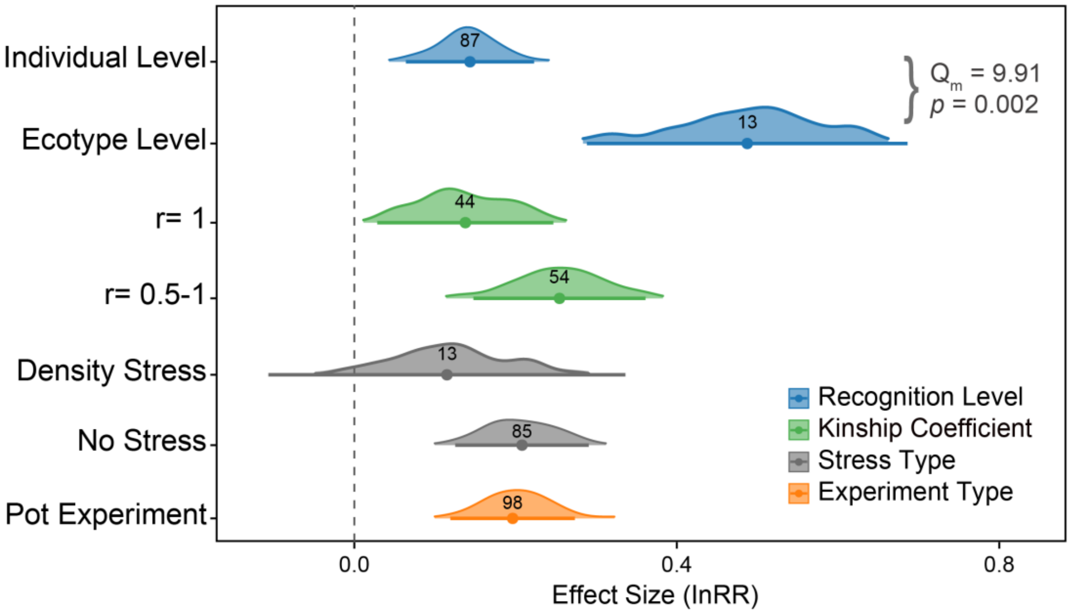

Figure S2. Biotic and abiotic factors influencing leaf area responses during kin coexistence in plants. Kin recognition levels are indicated in blue. The kinship coefficient *r* is shown in green. Stress types are represented in gray, while experimental types are shown in orange. Digitally represented observations.

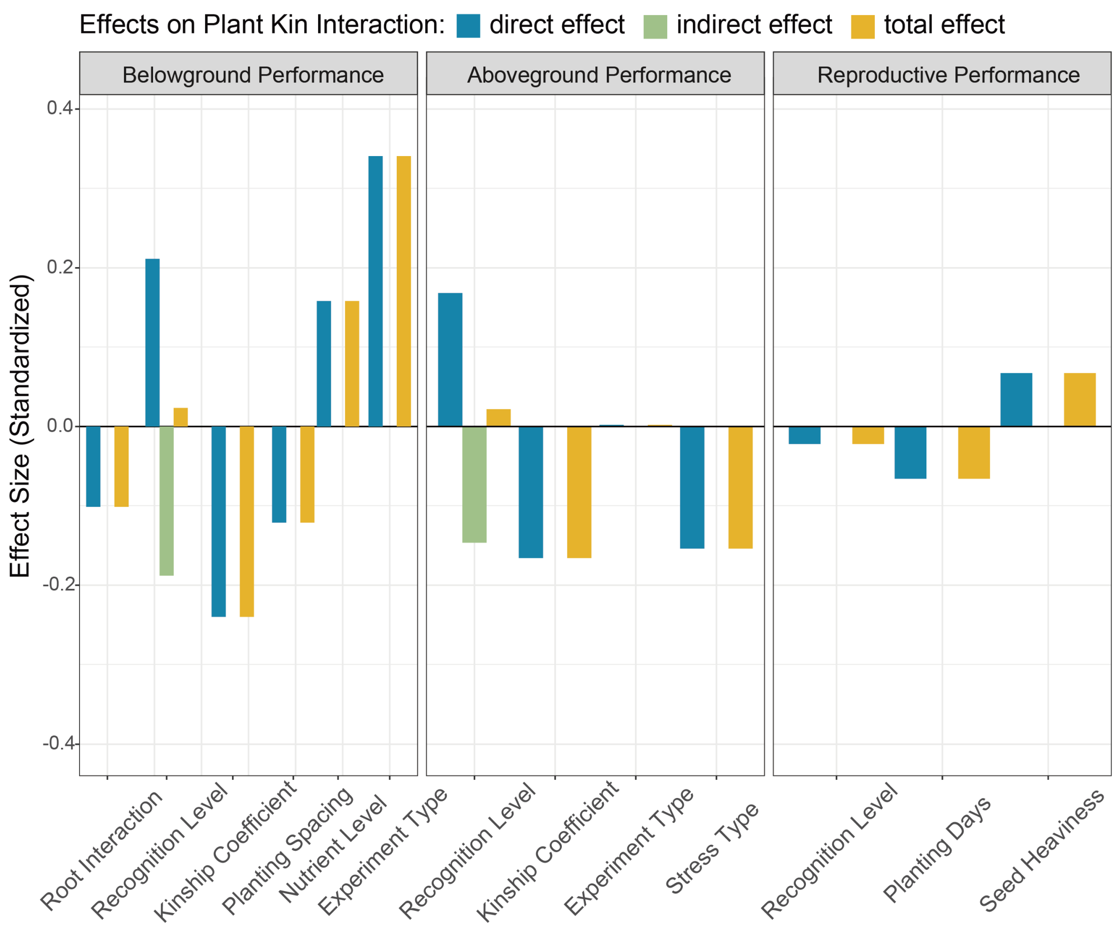

Figure S3. Standardized effect sizes of biotic and abiotic factors.

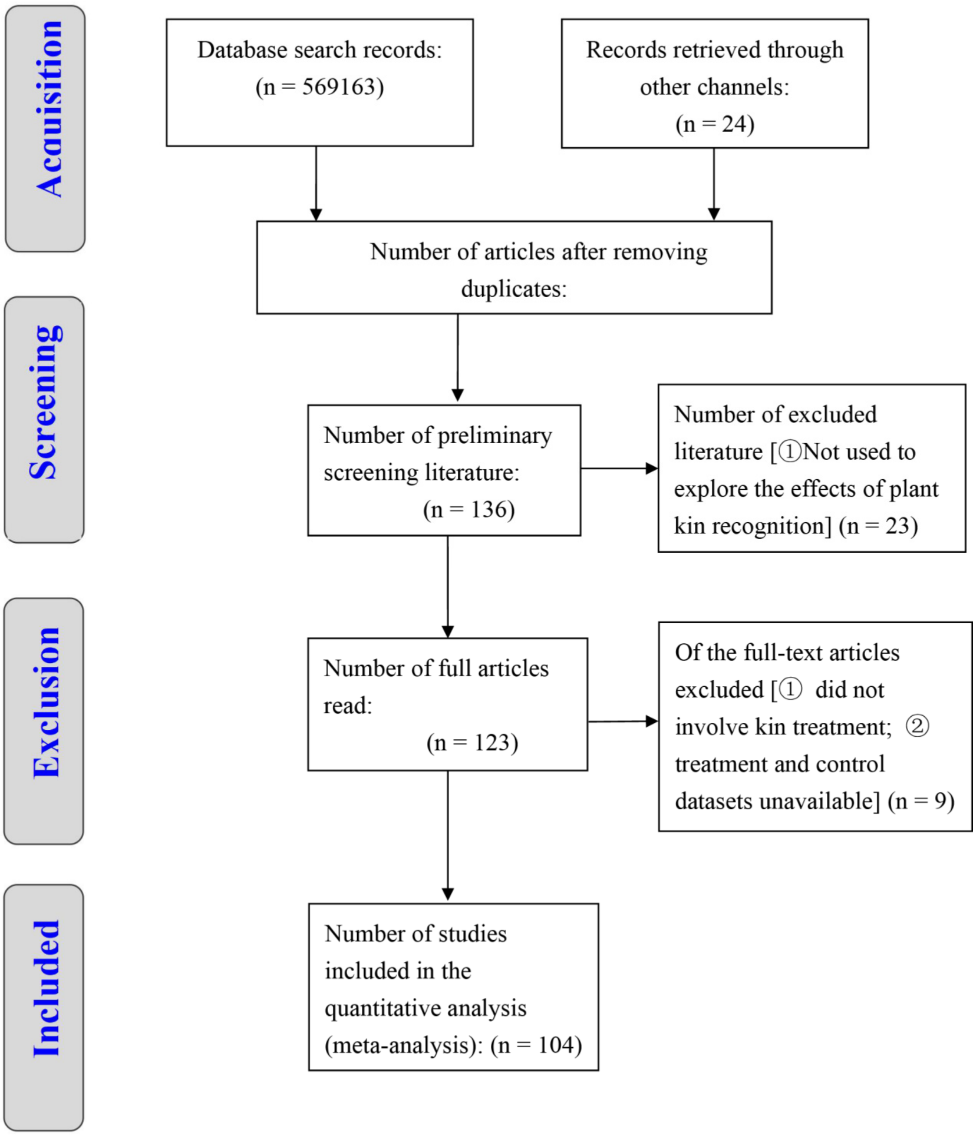

Figure S4. The literature screening process for the meta-analysis included in this study.

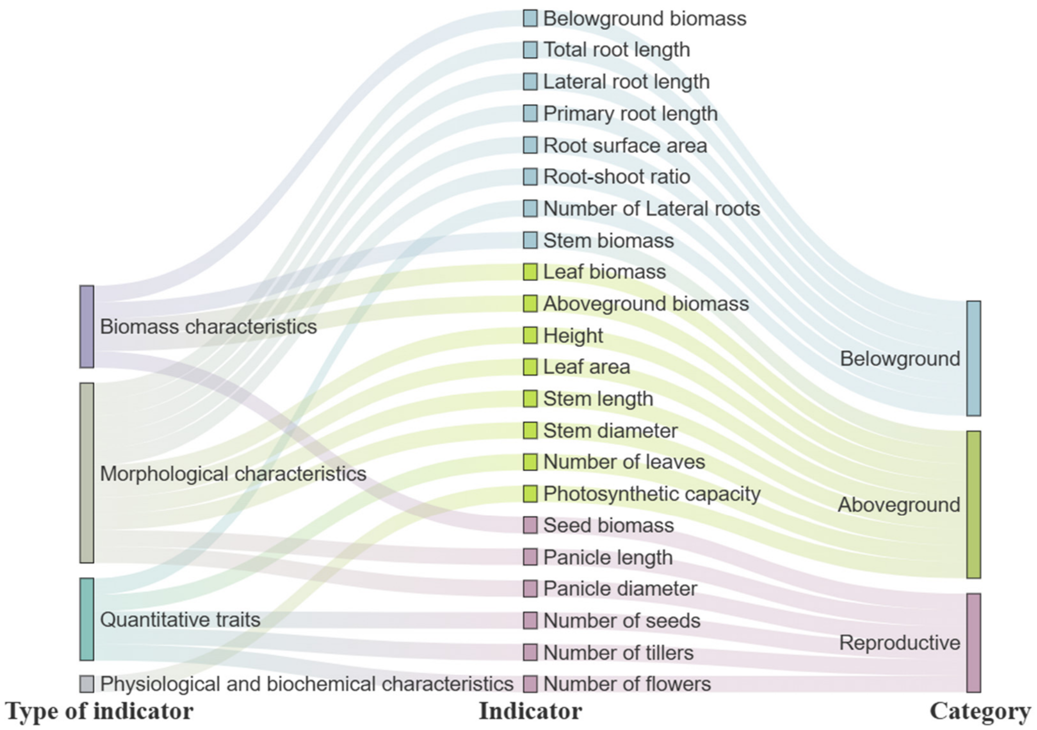

Figure S5. Classification of response metrics for kin recognition in plants.

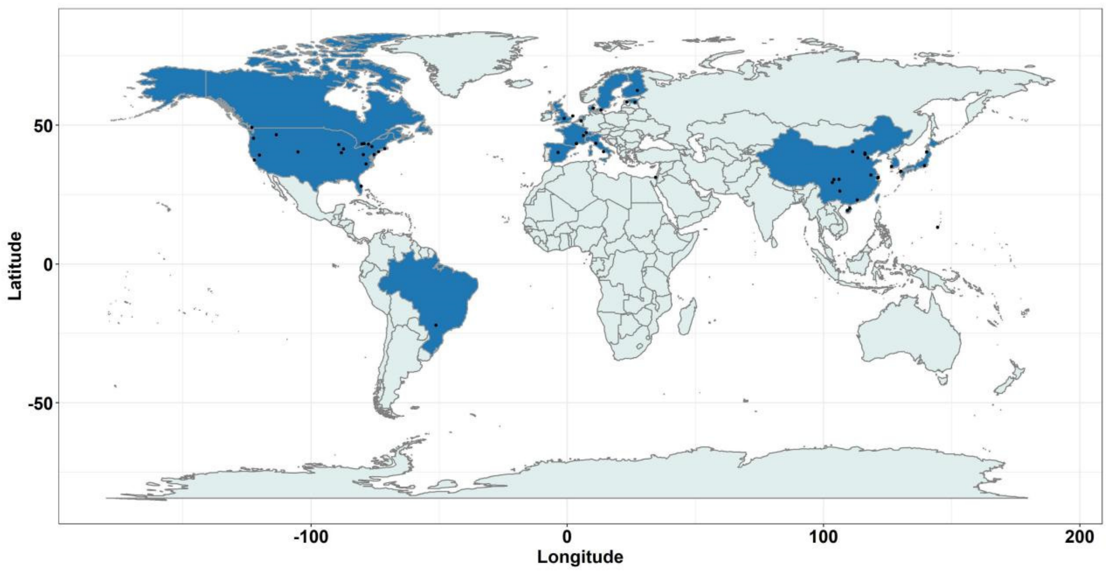

Figure S6. Study locations included in this meta-analysis. Black dots represent the latitude and longitude of the study sites, while the blue areas correspond to the countries associated with those coordinates.

Table S1. Study materials included in the literature on plant kin recognition and their aboveground, below-ground, and reproductive performance when coexisting with kin.

	Order	Family	Genus	Species	Belowground (InRR)	Aboveground (InRR)	Reproduction (InRR)
1	Poales	Poaceae	Distichlis	*Distichlis spicata*	0.2059	-	-
2	Poales	Poaceae	Triticum	*Triticum aestivum*	-0.0395	-0.0024	-0.009
3	Poales	Poaceae	Aegilops	*Aegilops triuncialis*	-0.0236	-	-
4	Poales	Poaceae	Triplasis	*Triplasis purpurea*	-	-0.1508	0.1401
5	Poales	Poaceae	Elymus	*Elymus sibiricus*	-0.0858	-	-
6	Poales	Poaceae	Leymus	*Leymus chinensis*	0.087	-0.0172	-0.2934
7	Poales	Poaceae	Lolium	*Lolium perenne*	-0.1318	-0.0465	-0.0139
8	Poales	Poaceae	Lolium	*Lolium multiflorum*	-0.3598	-	-
9	Poales	Poaceae	Setaria	*Setaria italica*	0.0775	0.0161	0.0522*
10	Poales	Poaceae	Sorghum	*Sorghum bicolor*	0.0277	0.0375	0.0358
11	Poales	Poaceae	Deschampsia	*Deschampsia caespitosa*	0.0661	-0.0659	-
12	Poales	Poaceae	Oryza	*Oryza sativa*	-0.1861***	0.0514***	0.1564***
13	Poales	Poaceae	Echinochloa	*Echinochloa crus-galli*	-0.1296***	0.2141*	0.1353
14	Poales	Poaceae	Zea	*Zea mays*	-0.013	0.0029	-
15	Poales	Poaceae	Buchloe	*Buchloe dactyloides*	-0.3277**	-	-
16	Asparagales	Iridaceae	Belamcanda	*Belamcanda chinensis*	-	-0.0239	-
17	Caryophyllales	Phytolacca-ceae	Phytolacca	*Phytolacca americana*	0.0368	0.0349	-
18	Caryophyllales	Amaran-thaceae	Portulaca	*Alternanthera philoxeroides*	0.1049***	0.0528***	-
19	Ericales	Ebenaceae	Diospyros	*Diospyros morrisiana*	-0.0061	-0.0367	-
20	Plantaginales	Plantaginaceae	Plantago	*Plantago lanceolata*	-	-	0.4166**
21	Plantaginales	Plantaginaceae	Plantago	*Plantago coronopus*	-	0.0512	-
22	Rosales	Fabaceae	Stylosanthes	*Stylosanthes guianensis*	-0.0415	0.039	-
23	Rosales	Fabaceae	Cotoneaster	*Caesalpinia pulcherrima*	-	-0.0673	-
24	Rosales	Fabaceae	Medicago	*Medicago lupulina*	-	-	0.3114

25	Rosales	Fabaceae	Medicago	*Medicago minima*	-0.0026	-	-
26	Rosales	Fabaceae	Stylosanthes	*Stylosanthes guianensis*	-0.112**	0.0441	-
27	Ranunculales	Balsaminaceae	Impatiens	*Impatiens balsamina*	-	0.0242	-0.0582
28	Ranunculales	Balsaminaceae	Impatiens	*Impatiens pallida*	0.0516	-0.0028	-
29	Salicales	Salicaceae	Populus	*Populus tomentosa*	0.3775	-0.0238	-
30	Salicales	Salicaceae	Populus	*Populus cathayana*	0.2812**	0.0345	-
31	Solanales	Convolvu-laceae	Ipomoea	*Ipomoea hederacea*	0.1819	-	0.0072
32	Solanales	Solanaceae	Solanum	*Lycopersicon lycopersicum*	-	0.0202	-
33	Saxifragales	Crassulaceae	Kalanchoe	*Kalanchoe daigremontiana*	0.2445*	0.0444	0.1896**
34	Lamiales	Lamiaceae	Stachys	*Stachys annua*	-	0.1085	-
35	Lamiales	Lamiaceae	Glechoma	*Glechoma hederacea*	-0.0029	-0.0491	-
36	Lamiales	Lamiaceae	Glechoma	*Glechoma longituba*	-0.2526***	-	-
37	Lamiales	Convolvu-laceae	Cuscuta	*Cuscuta europaea*	-	-0.0968	-
38	Malvales	Malvaceae	Abutilon	*Abutilon theophrasti*	-	0.1286*	-
39	Fagales	Fagaceae	Fagus	*Fagus crenata*	-	-0.0203	-
40	Fabales	Fabaceae	Pisum	*Pisum sativum*	0.0504	0.2941***	0.2761**
41	Fabales	Fabaceae	Glycine	*Glycine max*	-0.134***	-0.0994***	0.3869
42	Fabales	Fabaceae	Lupinus	*Lupinus angustifolius*	-0.1683	0.0993**	-0.17***
43	Asterales	Euphorbiaceae	Mallotus	*Mallotus japonicus*	-0.1178	-0.0438	-
44	Myrtales	Onagraceae	Oenothera	*Oenothera biennis*	-	-0.0296	-0.0614
45	Myrtales	Myrtaceae	Eucalyptus	*Eucalyptus urophylla*	-0.0744	0.2501	-
46	Brassicales	Brassicaceae	Arabidopsis	*Arabidopsis thaliana*	-0.1477***	0.1493***	0.1074***
47	Brassicales	Brassicaceae	Cakile	*Cakile edentula*	-0.1118***	0.1914	-
48	Brassicales	Brassicaceae	Capsella	*Capsella bursa-pastoris*	-	-0.0633	-
49	Brassicales	Brassicaceae	Moricandia	*Moricandia moricandioides*	-	0.0143	0.2777
50	Asterales	Asteraceae	Helianthus	*Helianthus tuberosus*	-0.1418**	-0.001	0.1617**
51	Asterales	Asteraceae	Taraxacum	*Taraxacum platycarpum*	-0.1301	-0.1359	-
52	Asterales	Asteraceae	Taraxacum	*Taraxacum officinale*	0.0155	-0.0512	-

53	Asterales	Asteraceae	Xanthium	*Xanthium italicum*	0.0736	0.27	-
54	Asterales	Asteraceae	Ambrosia	*ambrosia artemisiifolia*	-	-0.0415	-
55	Cycadales	Cycadaceae	Cycas	*Cycas edentata*	-0.2594*	-0.0052	-0.0701

Notes: *InRR:* Log response ratio, calculation method can be found in the Materials and Methods section. *InRR* of " - " indicates missing data for the species when coexisting with kin.

Table S2. Levels of interfering factors included in the meta-analysis of kin recognition in plants

Categories of Explanatory Variables		Levels of categorical variables					
		Level 1	Level 2	Level 3	Level 4	Level 5	Level 6
Biotic Factors	Root interaction	Contact	Separation	Semi-separa-tion			
	Kinship Coefficient *r*	1	0.5-1	0.5	0.25-1	0.25	
	Recognition Level	Individual Level	Variety Level	Ecotype Level			
	Main Pollination	Self-pollina-tion	Cross-pollina-tion	Mixed-mode Pollination			
	Selfing Rate	0-50%	51 % -95 %	96% - 100%			
	Photosynthesis Path-way	C3	C4	CAM			
	Main Reproduction	Sexual	Asexual	Both			
Abiotic Factors	Planting Spacing	(Continuous variables)					
	Nutrient Level	Low Nutrient	Normal Nutri-ent	High Nutrient			
	Experiment Type	Pot	Agar	Field	Hydroponic		
	Stress Type	No Stress	Nutrient	Density	Drought	Heavy Metal	Weed
	Pot Size	(Continuous variables)					
	Planting Days	(Continuous variables)					
	Application value	Cultivated	Wild				

Notes: (1) Root interaction: Root contact indicates direct contact, while root semi-separation involves nylon membranes or root exudate treatments; root isolation uses plastic membranes or separate growth. (2) Kinship Coefficient *r*: *r*=1 indicates that the related plants are clones. *r* = 0.5-1 signifies a self-fertilizing reproductive system with complete sibs. *r* = 0.5 represents the full siblings of outcrossing. *r* = 0.25-1 indicates an inbreeding reproductive system with biparental inbred sibs, while *r*=0.25 denotes an outcrossing reproductive system with half sibs. (3) Recognition level: Kin recognition levels are divided into three tiers. The individual level refers to sibling offspring as kin. The varietal level refers to closely related varieties as kin, while the ecological type level refers to kin within the same ecological type or biotype.
